# Cartilage breakdown in microgravity—a problem for long-term spaceflight?

**DOI:** 10.1038/s41536-017-0016-1

**Published:** 2017-04-11

**Authors:** Jamie Fitzgerald

**Affiliations:** Bone and Joint Center, Department of Orthopedic Surgery, Henry Ford Hospital System, Integrative Biosciences building, 6135 Woodward Ave, Detroit, MI 48202 USA

From the early days of spaceflight when humans first escaped the gravitational pull of Earth, it became clear that the unloading environment of microgravity (10^−6^G) had an impact on the human skeleton. The musculoskeletal system is acutely sensitive to changes in the biomechanical environment and prolonged exposure to microgravity causes bones to demineralize, and skeletal muscles to lose mass and strength.^1–5^ The lost tissue appears to be restored following return to normal gravity, although long-term studies have identified incomplete recovery of bone mineral density and architecture and permanent damage to bone remains a concern.^1, 2^

The musculoskeletal system is more than just bone and skeletal muscle and the effects of microgravity on other biomechanically sensitive elements are largely unknown. Of particular interest are articulating synovial joints, which are precisely engineered to accommodate large tensile and compressive biomechanical forces and, simultaneously, facilitate smooth mechanical action. Synovial joints are composed of articular cartilage integrated with sub-chondral bone, meniscal fibro-cartilage, tendon and several ligaments. These tissues are bathed in synovial fluid and enclosed in a fibrous capsule. Damage to any of these functionally interconnected elements lead to joint instability and compensatory connective tissue changes that can result in joint degradation. For the affected individual, these changes are often accompanied by progressively worsening pain, loss of mobility, and eventually, osteoarthritis (OA).^6, 7^ It is generally accepted that once the collagen II network in articular cartilage is broken down following proteoglycan degradation, restoration of authentic cartilage is no longer feasible. This is probably due to the lack of resident blood vessels, lymphatic vessels, and nerves, and access to mesenchymal or circulating stem cells. From a clinical perspective, no effective clinical options exist for the amelioration of late-stage OA and options for patients remain limited to symptomatic treatment until becoming candidates for total joint replacement. In recent years, treatments that attempt to repair or restore the cartilage lesion have started to be developed to slow or stop the progression towards OA but, in general, these are unsatisfactory with variable and unpredictable results.^8^ Given the poor regenerative capacity of cartilage, any microgravitational degradation would compromise flight crew mobility with the potential to negatively impact mission activities. Importantly, this damage may compromise the long-term joint health of flight personnel.

Is there evidence that biomechanical unloading impacts the joint? Data from terrestrial hind-limb unloading (HLU) animal experiments and human bed-rest studies demonstrate that reduced mechanical forces associated with joint unloading and immobilization leads to the proteoglycan loss in articular cartilage.^9–12^ Together, the joint trauma and unloading studies support the notion that a normal range of mechanical forces are critical for maintaining healthy joints. Interestingly, consistent with this hypothesis is the finding that active exercise prevented cartilage degradation in rats subjected to HLU^13^ suggesting that exercise may be useful as a countermeasure to minimize joint pathology in microgravity. With the goal of minimizing long-term musculoskeletal problems, pre-flight and post-flight physiotherapy and conditioning regimes to complement mid-flight exercise are being considered.^14^

If biomechanical unloading on Earth leads to cartilage breakdown, is there evidence that exposure to microgravity causes joint degradation? An early microgravity study reported smaller chondrogenic pellets, less proteoglycan synthesis and reduced dynamic stiffness of three-dimensional engineered cartilage constructs grown for 4 months on the Russian MIR craft.^15^ More recently, experiments using simulated microgravity,^16, 17^ and on parabolic flights, where repeated but short duration periods of microgravity are possible, have been conducted.^18, 19^ In studies on cultured human chondrocytes, parabolic flight resulted in upregulation of cytoskeletal network genes and proteins suggesting even with short duration microgravity, cells respond by reorganizing the cytoskeleton.^18^ Cytoskeletal reorganization were also reported in simulated microgravity experiments^16, 17^ as was reduction in extracellular matrix production and mineralization.^20, 21^ Our studies indicate that extended exposure to microgravity results in articular cartilage proteoglycan loss in mice (m/s in preparation). What about in humans? Cartilage degradation products would be present in synovial fluid, urine and blood of flight personnel. While elevated levels of bone resorption markers and calcium metabolism in body fluids of flight personnel have been reported,^22^ there are no studies that focused on cartilage breakdown products released by damaged joints, such as carboxy-terminal telopeptides of Type II Collagen. This deficiency can be addressed by sampling the stored fluids of astronauts for cartilage biomarkers.

If cartilage loss in microgravity does occur, as seems likely based on unloading studies on Earth and our mouse findings, several fascinating questions arise.

1. Degradation mechanisms—trauma vs. HLU vs. microgravity: Does the microgravity-induced cartilage breakdown occur via the same mechanisms as in the HLU model? If so, then HLU can be used to simulate the effects of microgravity. However, the possibility of a compounding effect of microgravity in addition to the unloading cannot be discounted. Similarly, are there common mechanistic steps between unloading and trauma that lead to cartilage destruction? The existence of a common response would suggest that findings from microgravity studies could inform mechanisms of OA with the potential to reveal new therapies.
2. Biochemical threshold for tissue restoration: Are there specific biochemical or molecular thresholds that need to be breached for tissue restoration to fail? Our studies suggest that microgravity is sufficient for reversible proteoglycan loss in mouse femoral cartilage. In these studies, the lack of surface fibrillation and damage suggests that the collagen network is intact and that the point-of-no-return has not yet been reached. Microgravity studies on joint tissues could be used to determine the relationship between cellular and molecular events and the potential for restorative tissue repair.
3. Gravitational threshold for tissue restoration: What is the minimal amount of gravity required to maintain healthy cartilage? If microgravity is detrimental to cartilage, would gravity at the surface of the moon (0.16 G), or Mars (0.38 G), be sufficient to maintain tissue regeneration pathways and prevent uncontrolled cartilage destruction? Efforts are underway to explore the use of fractional gravity generated by centrifugal forces to counteract the detrimental effects of microgravity. The application of partial gravity on long duration spaceflight may be the best countermeasure for a host of microgravity-induced pathologies.
4. Radiation and tissue regeneration: Does the increased radiation exposure of spaceflight affect tissue healing? The Earth’s magnetic field shields craft in low earth orbit (LEO) from significant radiation. However, beyond LEO and protection from radiation, the effects of radiation on biological systems may be significant. A recent report suggesting that radiation worsens cartilage loss caused by HLU suggests that radiation has a compounding effect on cartilage destruction.^10^

If the goal of long duration spaceflight is to be achieved then more research is needed into the effects of microgravity on cells, tissue explants, and whole animals on orbiting platform both in LEO, and beyond. Monitoring of joint health on the ISS can be achieved by fluid biomarker analysis and the expansion of existing imaging technologies^23^ to include cartilage. Understanding mechanisms of joint tissue damage in microgravity and the limitations governing consequent tissue repair on return to 1 G will provide insights into repair and regeneration processes for OA here on Earth.
